# Bacterial cellulose membrane functionalized with hydroxiapatite and anti-bone morphogenetic protein 2: A promising material for bone regeneration

**DOI:** 10.1371/journal.pone.0221286

**Published:** 2019-08-19

**Authors:** Fernanda Coelho, Maurício Cavicchioli, Sybele Saska Specian, Raquel Mantuaneli Scarel-Caminaga, Letícia de Aquino Penteado, Alexandra Ivo de Medeiros, Sidney José de Lima Ribeiro, Ticiana Sidorenko de Oliveira Capote

**Affiliations:** 1 Department of Morphology, São Paulo State University (UNESP), School of Dentistry, Araraquara, São Paulo, Brazil; 2 Department of General and Inorganic Chemistry, São Paulo State University (UNESP), Institute of Chemistry, Araraquara, SP, Brazil; 3 Department of Biological Sciences, São Paulo State University (UNESP), School of Pharmaceutical Sciences, Araraquara, São Paulo, Brazil; Kyoto Daigaku, JAPAN

## Abstract

Bone tissue engineering seeks to adequately restore functions related to physical and biological properties, aiming at a repair process similar to natural bone. The use of compatible biopolymers, such as bacterial cellulose (BC), as well as having interesting mechanical characteristics, presents a slow in vivo degradation rate, and the ability to be chemically modified. To promote better bioactivity towards BC, we synthesized an innovative BC membrane associated to hydroxyapatite (HA) and anti-bone morphogenetic protein antibody (anti-BMP-2) (BC-HA-anti-BMP-2). We present the physical-chemical, biological and toxicological characterization of BC-HA-anti-BMP-2. Presence of BC and HA components in the membranes was confirmed by SEM-EDS and FTIR assays. No toxic potential was found in MC3T3-E1 cells by cytotoxicity assays (XTT Assay and Clonogenic Survival), genotoxicity (Comet Assay) and mutagenicity (Cytokinesis-blocked micronucleus Test). The in vitro release kinetics of anti-BMP-2 antibodies detected gradually reducing antibody levels, reducing approximately 70% in 7 days and 90% in 14 days. BC-HA-anti-BMP-2 increased *SPP1*, *BGLAP*, *VEGF*, *ALPL*, *RUNX2* and *TNFRSF11B* expression, genes involved in bone repair and also increased mineralization nodules and phosphatase alcalin (ALP) activity levels. In conclusion, we developed BC-HA-anti-BMP-2 as an innovative and promising biomaterial with interesting physical-chemical and biological properties which may be a good alternative to treatment with commercial BMP-2 protein.

## Introduction

Bone regeneration is a complex multiple event process that includes necrotic bone tissue and clot reabsorption subsequent to trauma. There is the concomitant development of an inflammatory process that facilitates the release of growth factors that assist in cell differentiation, and consequently, in the formation of bone tissue [1].

In most cases, the bone regeneration process runs without complications (90 to 95%) [2]. However, prolonged bone defects following trauma or resection of cancer or unbound fractures may require more sophisticated treatment. In these cases, a combination of bone substitutes, such as biomaterials, with living cells/tissues or the use of these biomaterials alone may be appropriate [3].

Cellulose is the most abundant biopolymer and is present in a wide variety of living species, being obtained mainly from trees. It can also be obtained from the bacterium *Gluconacetobacter xylinus*, which produces lignin-free and hemicellulose-free nanobacterial cellulose in a three-dimensional hierarchical network composed of much thinner microfibrils of nanometeric size from 3.0 to 3.5 nm [4]. The cellulose synthesized by the bacteria is microcrystalline, it has superior water absorption capacity compared to vegetable cellulose, and it presents greater mechanical resistance when hydrated [5]. Unlike other types of synthetic membrane, bacterial cellulose membrane (BC) exhibits high resistance to chemical corrosion, is biocompatible, porous, and has mechanical resistance [6].

Hydroxyapatite (HA) is a hydroxylated calcium phosphate salt with a high degree of hardness, and is the major component of inorganic substance in bones and teeth. HA has biological properties such as biocompatibility and osteoconduction [7]. Bioceramics based on calcium phosphate (synthetic hydroxyapatite and tricalcium phosphate) resemble the inorganic components of bone tissue. These similarities make them prone to cell adhesion and neoformation of bone tissue, which makes them excellent materials to provide better bioactivity to bone repair materials [8].

Bone morphogenetic proteins (BMPs) belong to the *TGF-β* family and are considered the most important osteoinductive factor in demineralized bone matrix. The major function of BMPs is the recruitment of mesenchymal cells to the site of healing and differentiation into osteogenic lineage resulting in new bone formation [9]. It is believed that those pathways are activated by the binding of BMPs to membrane-specific binding receptor (BMP type 1 receptors (BMPR1) and BMP type 2 receptors (BMPR2)). This binding initiates signal transduction through the phosphorylation of different SMAD proteins and their nuclear translocation. SMADs also function as transcription factors. They control the expression of essential osteogenic genes involved in the proliferation of osteoblasts (Msx2), matrix synthesis (*RUNX2*, osteopontin-*SPP1*, alkaline phosphatase-*ALPL*) and inhibition of osteoclast differentiation (*TNFRSF11B*-osteoprotegerin) [10].

In addition to BMP/SMAD signaling, MAPK cascade represents an alternative non-canonical pathway for BMP-2 signal transduction. BMP-2 activates the p38, ERK1/2 and JNK1/2 signaling pathways to promote the expression and activation of a specific transcription factor related to *RUNX2*. *RUNX2* plays a key role in the osteoblastic differentiation of stem cells and directly stimulates the transcription of important downstream target genes, including those encoding osteocalcin (*BGLAP*), collagen type 1 (*COL1A1*) and osteopontin (*SPP1*) [11].

The use of BMPs was approved by the Food and Drug Administration, resulting in their rapid acceptance for bone fracture repair [12]. Literature recognizes that the use of BMP/INFUSE (Medtronic, USA) to perform spinal fusions contributes to greater perioperative and postoperative morbidity. However, production costs of clinical grade rhBMPs are high generating concern regarding their cost-benefit [13].

Most biological processes are guided by specific cellular mediators. The administration of only one exogenous growth factor would not present an ideal condition for wound healing [13]. As an alternative to exogenous rhBMP-2 administration, the use of immobilized anti-BMP-2 antibodies in scaffolds was studied for the capture of endogenous BMP-2. The captured endogenous BMP-2 would be able to induce osteogenic differentiation of osteoprogenitor stem cells. Such approaches are called Antibody Mediated Osseous Regeneration–AMOR [14].

Biomaterials are being developed using BC as the organic component, with inorganic compounds, such as HA, added to improve osteoconductive properties. This association makes BC-HA more similar to bone tissue [4]. The addition of anti-BMP-2 to BC-HA could promote greater osteoprogenitor stem cell differentiation and provide promising results for bone regeneration [14].

In our study, we hypothesized that the addition of anti-BMP-2 to BC-HA might represent a resourceful strategy to promote greater osteogenic differentiation of osteoprogenitor stem cells with promising results for bone regeneration. Here, we found that BC-HA-anti-BMP-2 enhanced the expression of several genes involved in bone repair, and increased both mineralization nodules and ALP activity suggesting that BC-HA-anti-BMP-2 is an innovative and promising biomaterial when combined with BMP-2 protein in bone regeneration therapy.

## Materials and methods

### Preparation of bacterial celulose/hydroxyapatite membranes (BC-HA)

*Acetobacter xylinum* was cultured in medium containing 50 g/L (m/v) glucose, 4 g/L yeast extract, 2 g/L anhydrous potassium phosphate anhydrous (KH_2_PO_4_), 0.73 g/L magnesium sulfate heptahydrate (MgSO_4_.7H_2_O), and 20 g/L ethanol at 30 °C. As a pre-inoculum, 10% *Acetobacter xylinum* and 90% of the culture medium were inoculated in flamed flasks and incubated at 30 °C for 24 hours. Then 100 mL of this solution was added to 13 cm diameter petri dishes and incubated for 5 days at 30 °C to produce cellulose. After this period, the membranes were washed with running water (3 days), treated with 300 mL of 0.1 mol L^-1^ NaOH at 80 °C for 30 minutes. The NaOH solution was renewed and left for another 30 minutes. The membranes were washed with distilled water for 3 days until neutral pH. Incorporation of HA into the BC membrane was performed according to Hutchens et al., [15] and Saska et al., [16]. Highly hydrated BC membranes (4 cm^2^ x 5 mm thick) were immersed in 20% ethanol at room temperature (25 °C) for 24 h. Alternating incubation cycles were performed in 20 mL of 0.05 mol·L^−1^ CaCl_2_ solution (pH 5.8) and 20mL of 0.1mol·L^−1^ Na_2_HPO_4_ solution (pH 9.1) at 25 °C. The membranes were dried at 50 °C for 3 days and sterilized by gamma radiation (20 kGy).

### Physical-chemical characterization of BC-HA composites

Scanning electron microscopy (SEM) images and energy dispersive X-ray spectroscopy (EDS) analysis were obtained from an FEG-SEM 7500F. After EDS analysis, the membranes were coated with 1 nm layer of gold for 60 s (3 kV and 15 μA). Fourier transforminfrared (FT-IR) spectra were obtained from dried powdered membranes on a Vertex 70, Bruker Fourier transform infrared spectrophotometer. The spectra were performed under the following conditions: percentage of absorbance (% A) with accumulation of 32 scans, with a resolution of 4 cm^-1^, in the absorption range of 400–4000 cm^-1^. The mechanical properties were determined in a TA-XT2 Texture Analyser (Stable Micro Systems) equipped with a ball tipped stainless steel probe (D = 2.5 cm). The parameters measured were puncture resistance (Rp), elongation (AP) and energy in the perforation per unit volume (Ep). These properties were verified in dry and moist samples, which were immersed in a solution with an ionic concentration similar to that of blood plasma, prepared according to Kokubo et al., [17]. Contact angle was measured by a Dataphysics model OCA-15 equipped with a CCD camera. The measurements were made by the dripping method using deionized water (surface tension: 72.30 mN/m); drops were dispensed by syringe with a 0.5 mm diameter needle and a volume of 10 μl at a drip speed of 1 μl.s^-1^ on to the membrane surfaces. Images of the captured droplets were analyzed using SCA20 software.

### Cell culture

MC3T3-E1 cells were seeded in α-MEM (1:1) culture medium, supplemented with 10% FBS and kanamycin (1%), and incubated at 37 °C with 5% CO_2_ and used at third passage. Treatments were in dupilicate and included negative and positive controls. Three independent experiments were conducted for each assay. MC3T3-E1 cells develop adhering to the cell culture flasks. After 24 h of seeding, the membranes were placed in contact with the culture medium in wells or cell culture flasks. The membranes have low density and float in the culture medium. MC3T3-E1 cells were kindly donated by Dr. Pedro Paulo Chaves de Souza, from UNESP University, Brazil.

### Toxicity assays

#### Cell viability (XTT assay)

Cell Proliferation Kit II (Roche Applied Science) was used. MC3T3-E1 cells (5×10^3^) were seeded in 48-well plates in a volume of 1 ml of α-MEM medium (1:1) supplemented with 10% FBS, and incubated at 37 °C in 5% CO_2_ for 7, 14, and 21 days. Treatments with BC and BC-HA membranes were for 24 h. Negative control (NC) contained only cells and culture medium, and positive control (PC) used doxorubicin hydrochloride (3.0 μg.mL^-1^) for 24 h. Each well was subsequently supplemented with 10% FBS with the exception of PC. After the incubation time, membranes were removed from each well and cells washed with PBS; 500 μL of DMEM without phenol red were added, followed by 60 μL of the XTT/electron solution (50:1). After 3 h incubation, a colorimetric spectrophotometer (VersaMax, Molecular Devices, Sunnyvale, CA) was used at 492 nm normalized at 690 nm.

#### Clonogenic survival assay

After 24h of seeding, 6×10^4^ cells were exposed to the treatments with BC and BC-HA membranes for 24 h. For PC, cells were treated with doxorubicin (0.3 μg.mL^-1^) for 4 h. The wells were washed with PBS and fresh medium was added. Cells (300 per 25 cm^2^ culture flask) were seeded and incubated at 37 °C, 5% CO_2_, for 7 days without culture medium changes. The colonies were fixed with methanol: acetic acid: water (1:1:8 v/v/v) for 30 minutes and stained with Giemsa 1:20 for 20 minutes. The number of colonies counted in the NC was considered as 100%. From this, survival fractions (SF) were obtained: SF = number of colonies counted in each treatment × 100/Number of colonies observed in NC.

#### Comet assay

The alkaline version of the Comet Assay was used according to the methodology described by Singh et al [18]. After 24 h of seeding, 50×10^3^ cells were exposed for 24 h to BC and BC-HA membranes and NC. PC used hydrogen peroxide (80 μmol.L^-1^) for 10 minutes. After centrifugation, the cell pellet was homogenized with 0.5% low melting point agarose, and the cell solution was dropped on to histological slides previously prepared with 1.5% normal melting point agarose. The slides were covered with coverslips and left at 4 °C for 10 minutes. After removal of the cover slip, the slides were immersed in a lysis solution for 2 hours protected from light at 4 °C. The slides were immersed in alkaline electrophoresis (pH 13) for 20 min for DNA denaturation, and the electrophoresis conducted at 43 V and 308 mA for 25 minutes. Slides were immersed in a neutralization buffer (Tris-HCl, pH 7.5) for 15 minutes at 4 °C. They were fixed in absolute ethanol for 3 min, and dried at room temperature. Staining was made with ethidium bromide (0.02 mg/mL). The slides were photographed using a fluorescence microscope and the images analized by an image analysis system (TriTek CometScore 1.5, 2006, Sumerduck, VA, USA). The percentage of DNA in Tail was obtained for each treatment, with 100 nucleotides analyzed for each treatment [19].

#### Cytokinesis-blocked micronucleus (CBMN) assay

Fenech [20] protocol was used with some modifications. 40×10^4^ MC3T3-E1 cells were seeded in 25 cm^2^ culture flasks containing 5 mL of α-MEM medium (1:1). After 24 h of seeding, cells were exposed to BC and BC-HA membranes, and NC for 24 h. For PC, cells were treated with doxorubicin (0.15 μg/mL) for 4 hours. Cytochalasin-B (CytB) was added to MC3T3-E1 at a final concentration of 25 μL of cytochalasin B (1mg/mL) for 48h to stop cytokinesis. The cells were washed with PBS, trypsinized, and the contents of the culture flasks transferred to a 15 mL tube and centrifuged for 7 minutes at 1000 rpm. The pellet was resuspended in ice cold solution (0.3% KCl) for 5 min. This cell suspension was centrifuged again and resuspended in 3 mL of methanol: acetic acid (3:1) fixative with four drops of 1% formaldehyde. After further centrifugation, 500 μL of the cell solution was dropped on to slides previously cleaned in methanol and kept in ice-cold distilled water. The slides were stained with 3% Giemsa solution diluted in phosphate buffer (0.06M Na_2_HPO_4_ and 0.06 M KH_2_PO_4_—pH 6.8) for 7 minutes, washed with distilled water and dried at room temperature. For the determination of the Nuclear Division Index (NDI), 500 viable cells with well preserved cytoplasm were counted using a Leica DM500 microscope. The NDI was calculated according to Eastmond and Tucker [21] using the formula: NDI = [M1 + 2(M2) + 3(M3) + 4(M4)]/N, where M1 to M4 is the number of cells with 1, 2, 3 and 4 nuclei, respectively; and N is the total number of viable cells. The frequency of binucleate cells with micronucleus (FBMN) and the total frequency of micronucleus (FMN) were analyzed in 1000 binucleated cells. The criteria used to identify micronucleus were based on Fenech [20].

### Immobilization of anti-BMP-2 monoclonal antibody on bacterial cellulose membranes and verification of in vitro antibody adsorption kinetics

Anti-BMP-2 antibody (Clone 3g7-Abnova, USA) was commercially purchased and immobilized on membranes according to Freire et al [14]. The antibody was diluted in PBS at a concentration of 25 μg/mL and incorporated by adsorption onto 25 mm diameter membranes for 24 h [14].

### Anti-BMP-2 adsorption evaluation

FITC-conjugated anti-mouse IgG2a antibodies were used as a strategy to evaluate the adsorption of the anti-BMP-2 antibodies into CB-HA membranes [14]. At several time-points (1, 3, 7, and 14 days), before adding the secondary antibody, membranes were left in PBS containing 10% FBS for 1 hour at room temperature. Imaging was performed using a fluorescence microscope and acquired images were analyzed for FITC intensity using ImageJ (NIH, Bethesda, MD, USA) software.

### Gene expression

MC3T3-E1 cells (5x10^3^) were seeded on 48-well plates. The cells were cultured with 1 mL of osteogenic medium composed of Dulbecco's modified medium (DMEM) containing 10% FBS, 1% penicillin-streptomycin (PS), 2M β-glycerophosphate, and 50 μg/mL ascorbic acid. The cultured MC3T3-E1 cells were submitted to different treatments as listed in Table 1 for 7, 14, and 21 days. After the treatments, total RNA from the MC3T3-E1 cells was extracted with Trizol (Invitrogen, Carlsbad, CA). Complementary DNA (cDNA) was synthesized by reverse transcription using a High Capacity cDNA Reverse Transcription (Invitrogen) kit with oligo(dT)20. Real-time quantitative PCR (qPCR) reactions were performed by the TaqMan system (Applied Biosystems, Foster City) to assess expression of the genes of interest: *SPP1* (Osteopontin, Cat No. Mm00436767_m1), *ALPL* (Alkaline Phosphatase: Cat. No. Mm00475834_m1), *TNFRSF11B* (Osteoprotegerin Cat. No. Mm01205928_m), *RUNX2* (Cat. No. Mm00501584_m1), *BGLAP* (Osteocalcin, Cat no. Mm00649782_gH) and *VEGF* (Vascular endothelial growth factor, Cat no. Mm01281449_m1). To normalize gene expression levels, Glyceraldehyde-3-phosphate Dehydrogenase *(GAPDH)* was used as a housekeeping gene (cat. No. Mm99999915_g1). Relative gene expression values of the genes of interest were analyzed using the Expression Suite software (Applied Biosystems), and fold change (2-^ΔCt^) results were presented in graphs.

**Table 1:** Materials evaluated in the Gene expression assay.

**MC3T3-E1 cells (G1)—NC**
**MC3T3-E1 cells + 200ng/mL BMP-2 protein (G2)—PC**
**MC3T3-E1 cells + BC-HA-anti-BMP-2 (G3)**
**MC3T3-E1 cells + BC-HA-anti-BMP-2 + 200ng/mL BMP-2 protein (G4)**
**MC3T3-E1 cells + BC-HA + 200ng/mL BMP-2 protein (G5)**

### Detection of mineral nodule formation by alizarin red

MC3T3-E1 cells (5x10^3^) were seeded in osteogenic medium using 90 mm x 15 mm Petri dishes in contact with BC-HA-anti-BMP-2 for 7, 10, and 14 days. Cells were washed with PBS, fixed in 4% formaldehyde for 30 minutes, and washed sequentially with PBS. They were stained with 40 mM Alizarin red pH 4.2, at room temperature for 30 minutes. For quantitative evaluation of mineral nodule formation, dye was diluted with 10% acetic acid solution and 10% ammonium hydroxide. Subsequently, 150 μl of this solution was transferred to a 96-well plate and the absorbance reading of the samples was performed by a spectrophotometer (VersaMax, Molecular Devices, Sunnyvale, CA) with a wavelength of 405 nm.

### ALP activity

MC3T3-E1 cells (5x10^3^) were seeded in osteogenic medium in 48-well plates in contact with BC-HA-anti-BMP-2 forr 7, 10, and 14 days. The release of thymolphthalein was assessed by the hydrolysis of thymolphthalein monophosphate substrate using a commercial kit (Labtest Diagnóstica, Belo Horizonte, MG, Brazil). Reaction absorbance was evaluated in a spectrophotometer (VersaMax, Molecular Devices, Sunnyvale, CA), using 590 nm wavelength and alkaline phosphatase activity, measured from the standard curve using thymolphthalein. Data were normalized by total protein content, which was determined by the Lowry method: proteins were initially extracted from each well by adding 1 mL/well of 0.1% sodium lauryl sulfate (Sigma) for 40 minutes at room temperature (RT). A quantity of 100 μL of the Lowry solution (Sigma) was added to 150 μL of the samples and incubated for 20 minutes at RT. After incubation, 50 μL of Folin & Ciocalteu's solution (Sigma) was added and incubated for 30 minutes at RT. Absorbance was evaluated at 595 nm by spectrophotometer (ELx 800, Bio-Tec). Total protein content was then calculated using a standard curve determined from bovine albumin and expressed in μg.mL^-1^.

### Statistical analysis

Statistical analysis was performed using Graphpad Prism 5.01. For parametric results, one-way ANOVA was applied followed by the Tukey test. Dunnett’s test was also used to compare treatments with NC. Non-parametric results used the Kruskall-Wallis test followed by Dunn’s test. Gene expression analysis used Two-way analysis of variance (ANOVA) followed by the Bonferroni test. The level of significance was 5%.

## Results

### Characterization of BC-HA membranes

**Fig 1:**
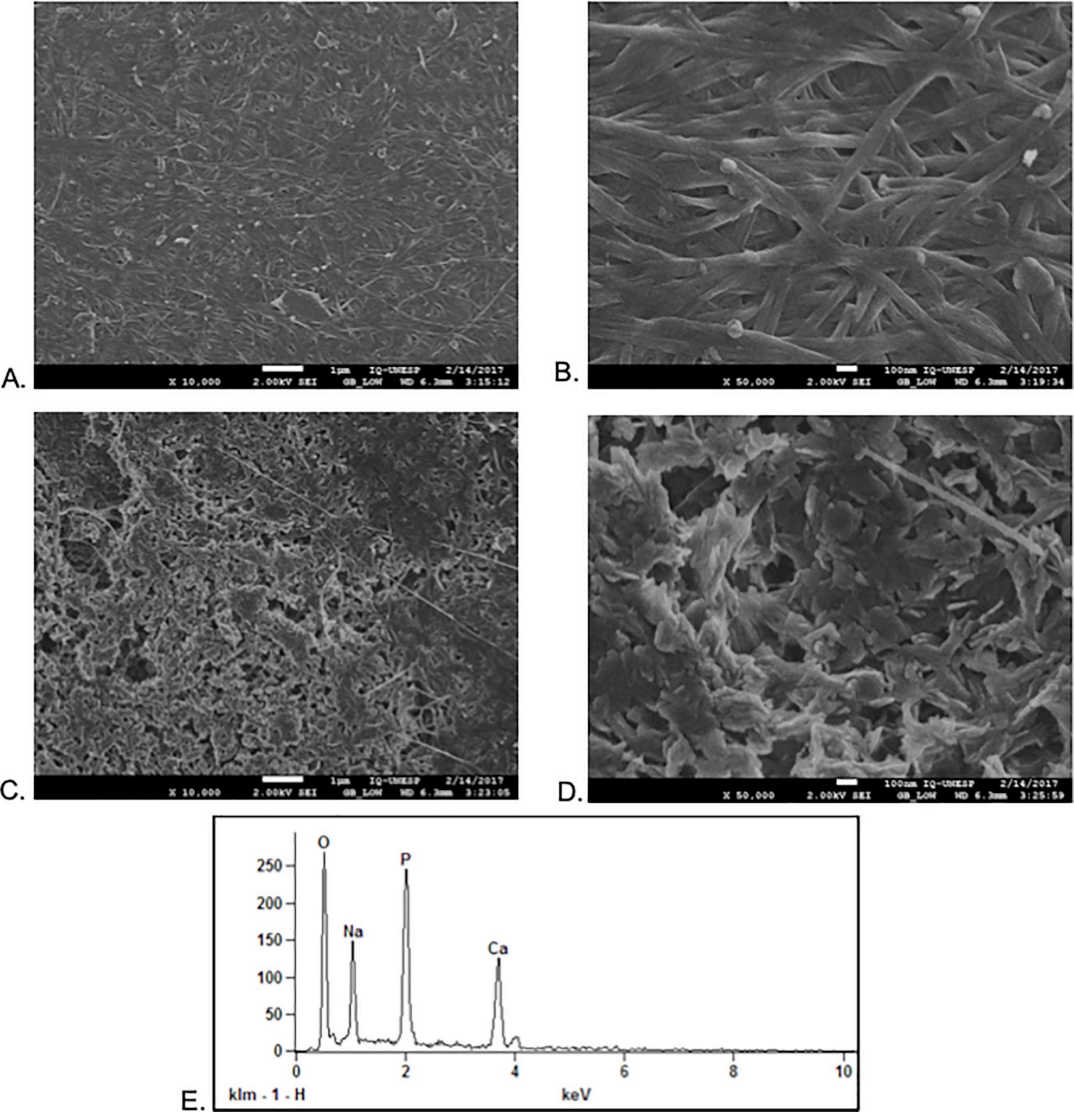
SEM images of the BC and BC-HA membranes. (a) and (b) BC surface at 10,000X and 50,000X, respectively; (c) and (d) BC-HA surface at 10,000X and 50,000X, respectively. Acceleration voltage was 2kV for these samples. (e). EDS spectrum from a typical membrane with surrounding HA crystals.

Scanning electron microscopy (SEM) images of BC and BC-HA are shown in Fig 1. The micrographs of the BC-HA membranes surfaces revealed nanometric HA crystals homogeneously covering the CB fibers, and pores regularly distributed on the surface of the membrane (Fig 1C and 1D). EDS data showed that qualitatively measured Ca/P molar ratios are in agreement with observed HA crystal morphology for BC-HA. Calcium phosphates have different solubilities and comparative extent of dissolution: OCP (Ca/P = 1.33) ß-tricalcium phosphate (ß-TCP) (Ca/P = 1.48), calcium deficient HA (CDHA)/P = 1.52) (Fig 1E). Regardig BC-HA, a Ca/P molar ratio was obtained between 1.49 and 1.54 with a mean of 1.52. This Ca/P molar ratio being related to a calcium deficient phosphate HA (CDHA). This difference reproduces the influence of the crystallographic properties of calcium phosphate, since HA is insoluble in body fluids.

**Fig 2:**
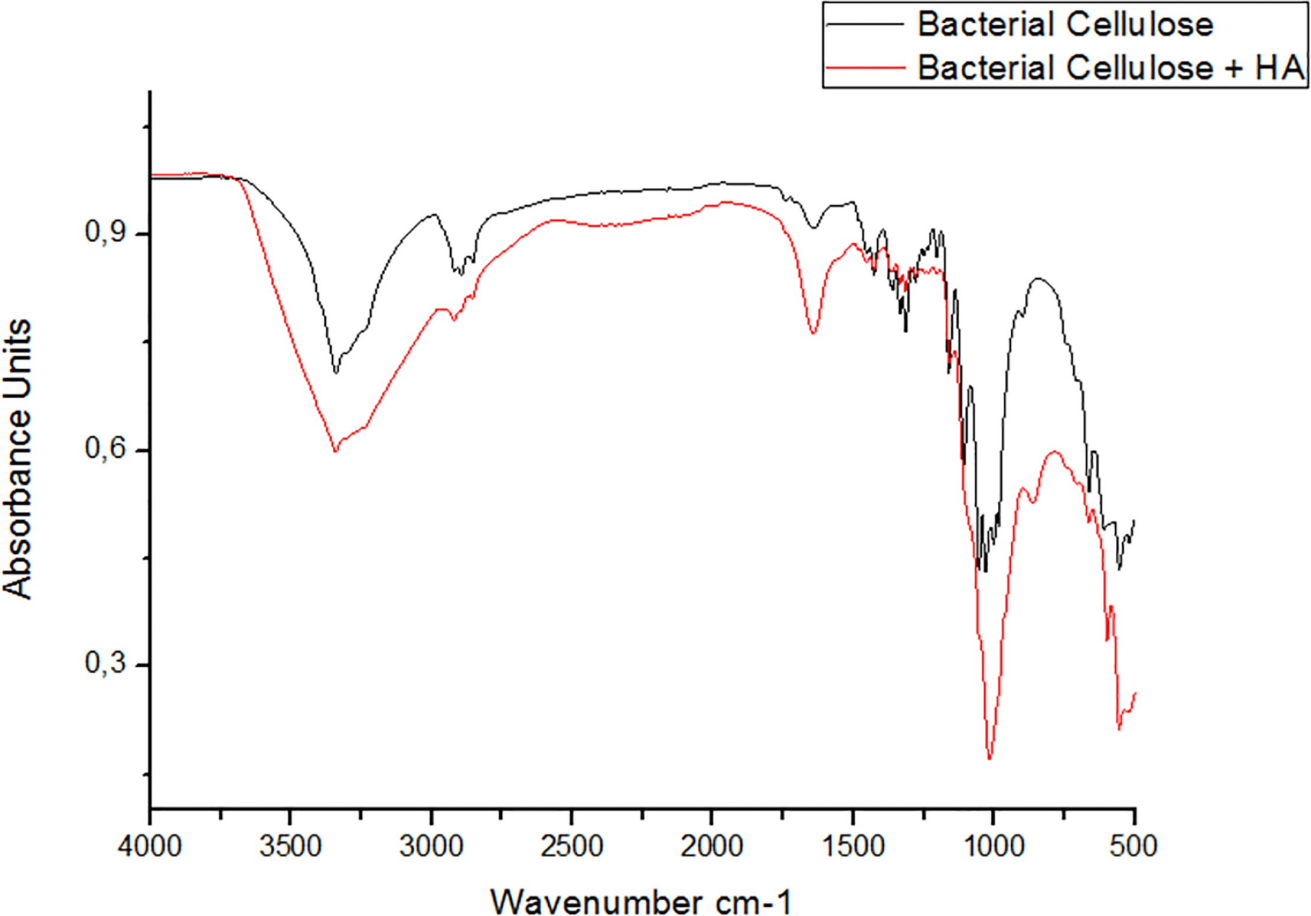
FTIR spectra of BC (black line) and BC-HA (red line).

FTIR spectra of dried BC membranes and BC-HA composite membranes are shown in Fig 2. The main attributes that characterize cellulose are: ~ 3450 cm^-1^—OH stretch; ~ 2900 cm^-1^ stretching CH of alkanes and asymmetric drawing CH_2_; 2700 cm^-1^—symmetrical drawing CH2; 1645 cm^-1^ OH deformation; 1432 cm^-1^ deformation CH_2_; ~ 1370 cm^-1^ deformation CH_3;_ ~ 1332 cm^-1^—OH deformation, and in the region of 1320–1030 cm^-1^ CO^17^ deformation.

The BC-HA membranes presented a spectrum with typical phosphate bands with apatite structure. Intensive bands were observed at 1093, 1020, 962, 570–600 cm^-1^ corresponding to the stretching of PO_4_^3-^ ions [15,22,23]. The low intensity bands located around 1428 and 838 cm^-1^ correspond to the stretching of CO_3_^2-^ ions [16]. The low intensity band around 3500–3200 cm^-1^ (OH stretch) observed in the CB spectra suffered a slight decrease in spectrum intensity in the BC-HA composite, suggesting a possible interaction between the HA crystals and the cellulose hydroxyl groups.

The mechanical properties of dried BC membranes and BC-HA composite membranes are shown in Table 2.

**Table 2:** Mechanical properties of membranes.

Membranes	(R_P_) (MPa)	(A_p_) (%)	(E_P_) (k J m^3^)
Dry BC	16.075 ± 0.721	0.942 ± 0.568	415.949 ± 48.248
Dry BC-HA	6.812 ± 3.229	0.622 ± 0.223	234.205 ± 31.598
Wet BC	23.465 ± 3.756	0.709 ± 0.111	670.067 ± 18.113
Wet BC-HA	7.888 ± 0.396	0.529 ± 0.200	148.875 ± 4.934

Rp: resistance to drilling; Ap: stretching during drilling; Ep: energy in drilling per unit volume.

The presence of hydroxyapatite in both dry and wet membranes revealed significantly less mechanically resistance (lower Rp values) compared to membranes without hydroxyapatite (p <0.0001; Tukey).

Ep results are in agreement with those of Rp for the BC membranes, since the membranes exhibiting the highest energy expenditure in perforation also showed higher Rp values, while the membranes with lower Rp showed the lowest Ep values. The parameters (Rp, Ep and Ap) used to evaluate the mechanical properties of BC membranes increased when they were moistened. This result reveals that the membranes became less fragile, and would possibly exhibit greater resistence in vivo.

The contact angle test performed to determine the hydrophilic properties of the composites before and after the immobilization of the anti-BMP-2 antibodies is shown in Table 3.

**Table 3:** Measured contact angle values for BC and BC-HA samples.

Membranes	Contact Angle (°) ± SD
BC	15.26 ± 0.45
BC-HA	2.1 ± 0.36
BC-anti-BMP-2	5.13 ± 0.20
BC-HA-ANTI-BMP-2	0.58 ± 0.51

Contact angle measurements for BC, BC-HA, BC- anti-BMP-2, BC-HA- anti-BMP-2 samples revealed that all surfaces of these samples were hydrophilic because they had a contact angle θ<90°. The presence of anti-BMP-2 in BC-anti-BMP-2 and BC-HA-anti-BMP-2 further decreased this angle, showing that they were slightly more hydrophilic.

### Toxicity assays

**Fig 3:**
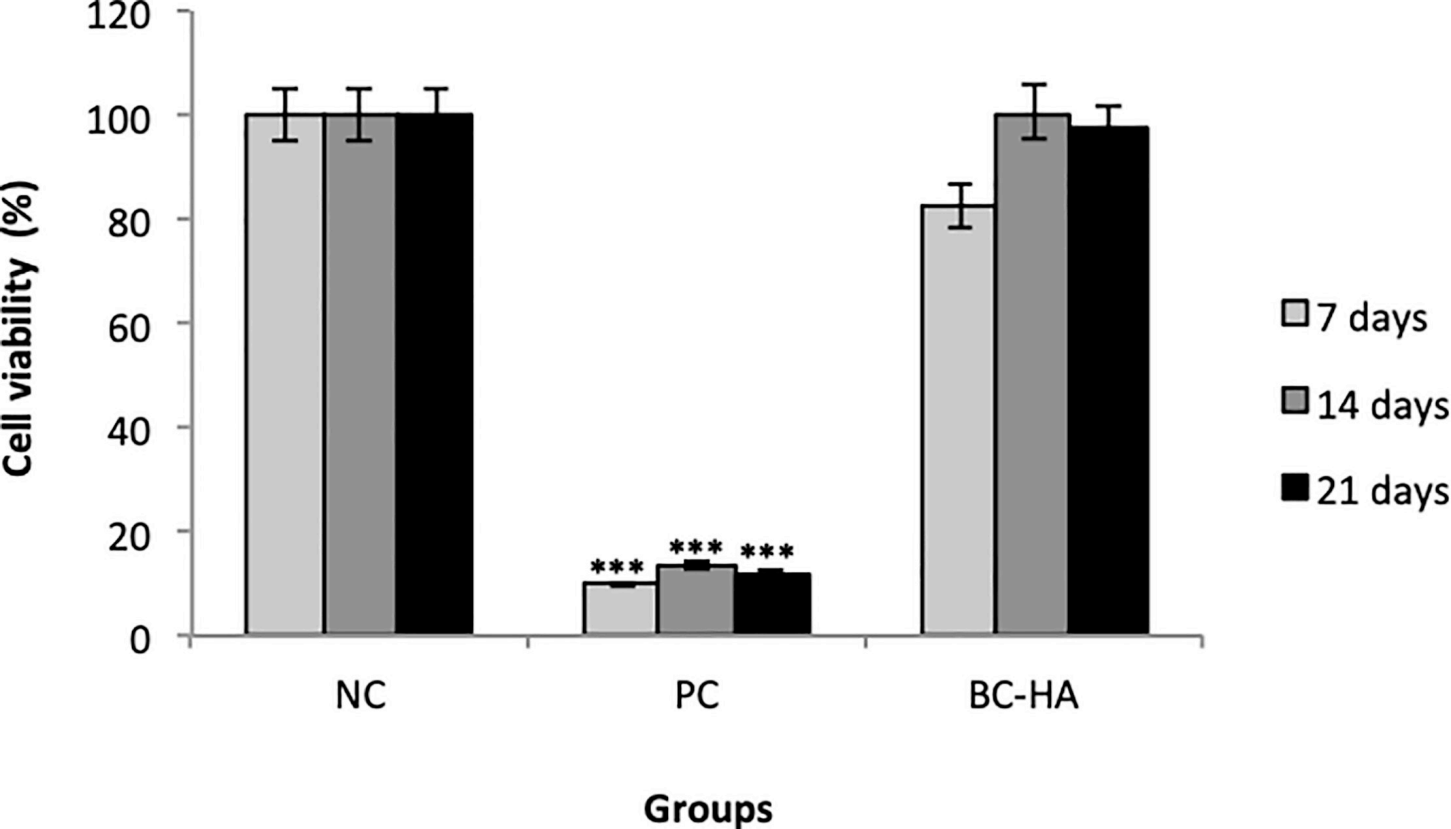
Cytotoxicity (cell viability) assessed by the XTT method. Columns indicate mean cell viability values. Bars indicate standard errors. The *** symbol indicates a statistically significant difference compared to NC (p<0.0001). Dunnett's test.

After 7, 14, and 21 days of contact with MC3T3-E1 cells, cell viability of the materials was assessed by XTT assay. BC-HA showed high cell viability (minimum of 100%), similar to NC (Fig 3).

**Fig 4:**
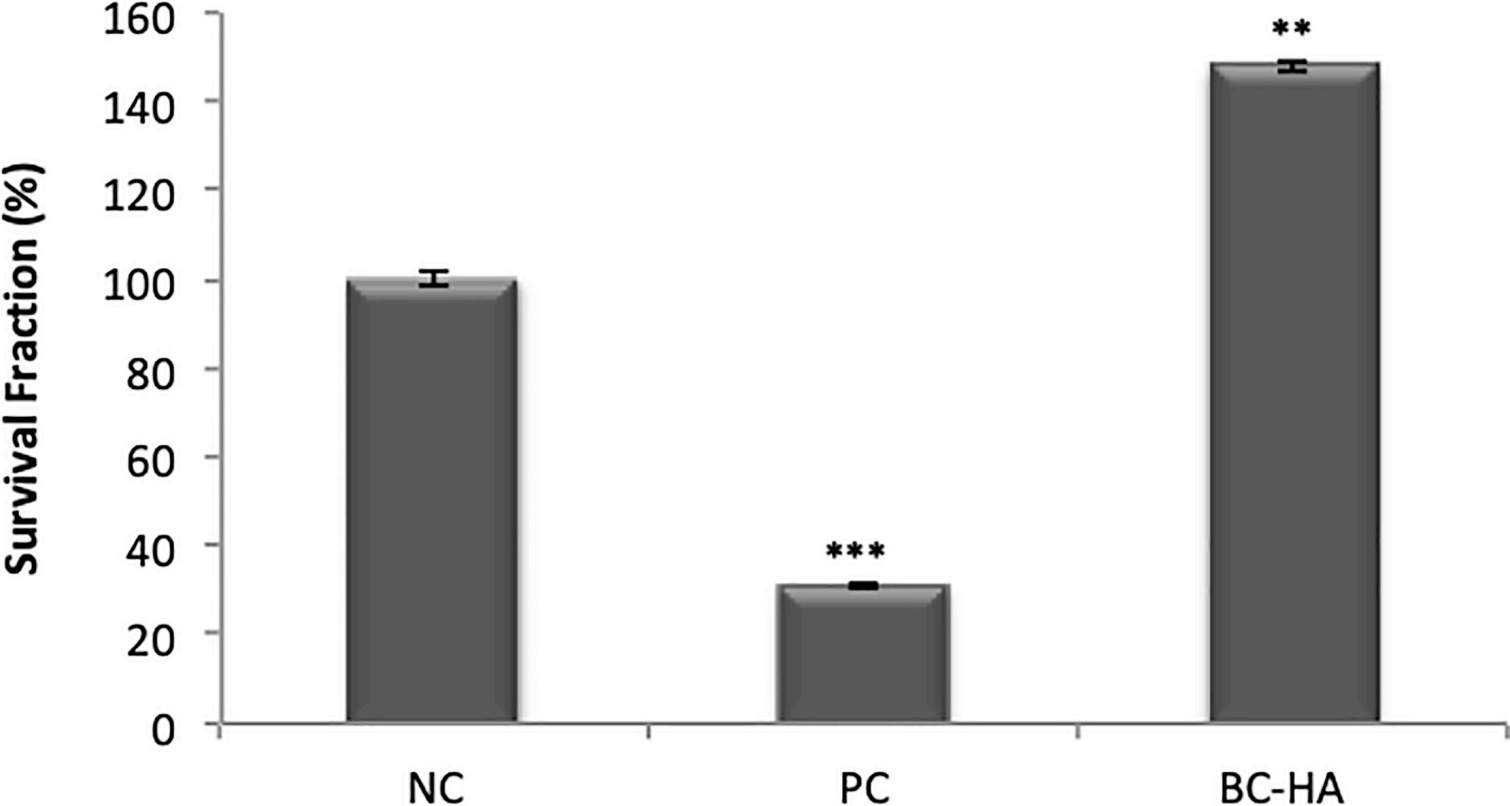
Cytotoxicity assessed by clonogenic survival assay. Columns indicate mean Survival Fraction (%). NC represents 100% survival fraction. Bars indicate standard error. The *** symbol indicates statistically significant difference compared to NC (p<0.0001). Dunnett's test.

Survival Fraction results from the Clonogenic Survival assay revealed that BC-HA presented a significantly different survival fraction to NC (Fig 4); this difference was due to an improvement in cell proliferative capacity (p <0.001; Dunnett).

Both cytotoxicity tests (XTT and Clonogenic Survival) showed similar results. As well as not being cytotoxic, BC-HA promoted higher cell viability (XTT method), and did not impair cell proliferative capacity (Clonogenic Survival).

**Fig 5:**
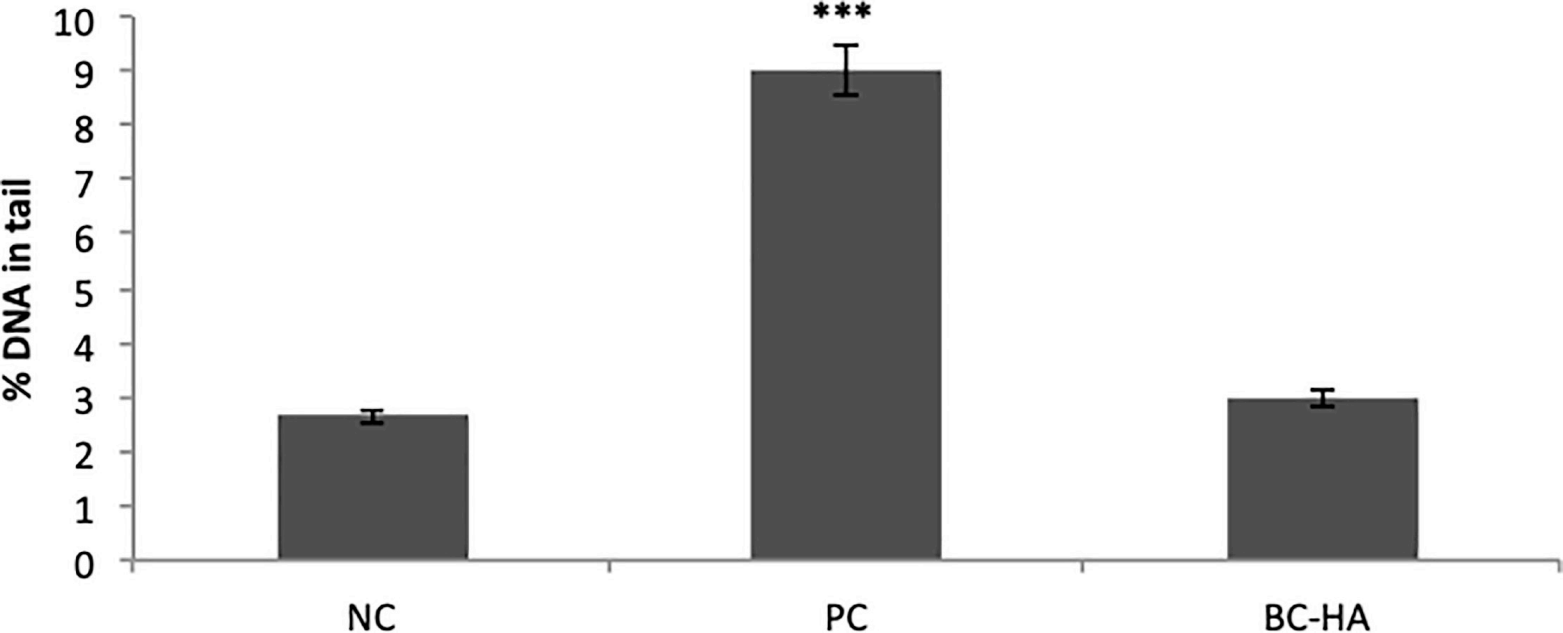
Genotoxicity assessed by the comet assay. Columns indicate mean percentage of DNA in the nucleoid tail. Bars indicate standard error. The *** symbol indicates statistically significant difference compared to NC (p<0.0001). Dunnett's test.

Analyzing the percentage of DNA in the tail by Comet Assay (Fig 5) revealed that BC-HA was not genotoxic compared to NC (p>0.05; Dunnett).

Complementing genotoxicity analysis, a mutagenicity test was performed using the Cytokinesis-blocked micronucleus (CBMN) assay. Results are shown in Table 4.

**Table 4:** Cytokinesis-blocked micronucleus assay results showing Mean and Standard Error values for Nuclear Division Index (NDI), Frequency of Micronucleus Binucleated Cells (FMNB), Frequency of Micronuclei (FMN), Nucleoplasmic Bridges (NB) and Nuclear Buds from each treatment with materials and controls.

**Group**	**NDI** **Mean±SE**	**FMNB** **Mean±SE**	**FMN** **Mean±SE**	**NB** **Mean±SE**	**Buds** **Mean±SE**
**NC**	1.68 ± 0.005	9.33 ± 0.919	11.00 ± 0.632	3.00 ± 0.365	4.33 ± 0.760
**PC**	1.57 ± 0.005^b^	56.00 ± 1.317^a^	95.3 ± 2.565^a^	19.33 ± 0.918^a^	13.67 ± 0.431^a^
**BC-HA**	1.66 ± 0.008	15.33 ± 0.55	17.00 ± 0.365	5.33 ± 0.557	3.66 ± 0.210

Mean and standard error (SE).

(a) indicates a statistically significant difference compared to NC (p<0.0001; Dunnett).

(b) = p<0.0001, Tukey.

Nuclear Division Index (NDI) showed that the cell division process was only nonuniform in PC compared to NC, as we observed a cytostatic effect by doxorubicin hydrochloride (p<0.0001; Tukey).

Evaluation of FBMN, FMN, NB and Buds showed that only PC was statistically different to NC. It can therefore be concluded that BC-HA showed no mutagenic potential in any of the parameters assessed for MC3T3-E1.

### Antibody in vitro adsorption tests and quantification

#### BMP-2 is incorporated at BC-HA membranes

**Fig 6:**
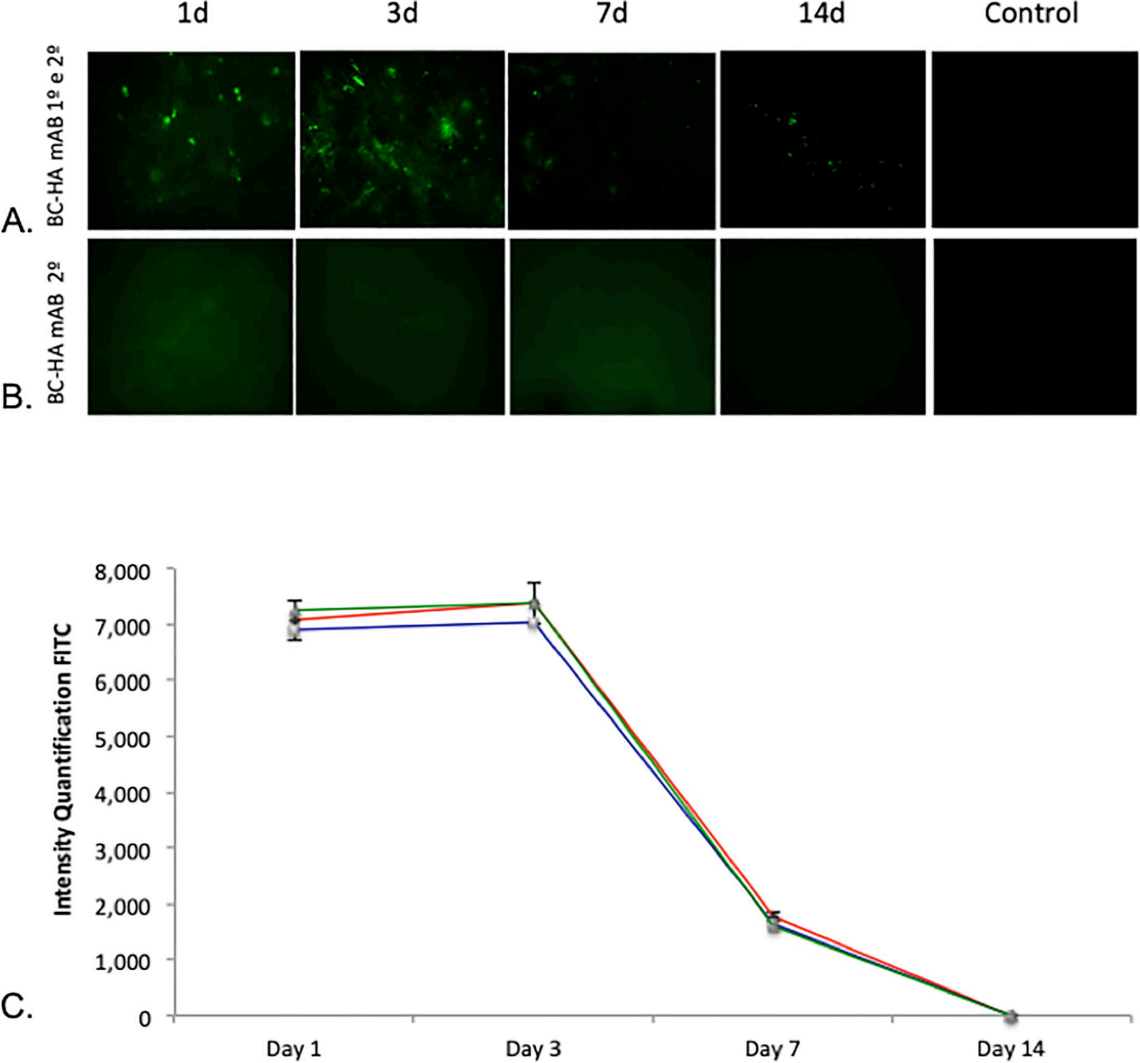
Characterization of the in vitro adsorption profile of BMP-2 on BC membranes. Anti-BMP-2 adsorption on BC-HA was evaluated by incubation with FITC-conjugated anti-mouse IgG2a at different times (1, 3, 7, and 14 days). (B) BC-HA incubated with FITC-conjugated anti-mouse IgG2a at different times (1, 3, 7, and 14 days). The Control column is represented by membranes incubated with secondary antibody alone. (C) Quantification of FITC intensity was performed by ImageJ software (NIH, Bethesda, MD, USA). The results from one experiment are presented.

An in vitro adsorption test was performed to evaluate the kinetics of BMP-2 adsorption onto BC membranes over time using a FITC-conjugated anti-mouse IgG2a against anti-BMP-2 mAb. Qualitative results demonstrated that anti-BMP-2 mAb was easily visble at day one, lasting until day 3, and then decreasing through Day 7 and 14 (Fig 6A and 6B). These results were quantified by FITC intensity using ImageJ (NIH) software (Fig 6C).

### Gene expression

The RNA extraction method was efficient in quantity and quality for all cell treatments, and amplification efficiency for all genes was similar and close to 100%.

It is worth taking into account that the G2, G4 and G5 groups contained both the BMP-2 protein (200 ng/mL) and α-MEM [24] culture medium. G1 demonstrated how constitutively expressed each gene was by stimulus from just the osteogenic medium. G2 represented the PC because it contained BMP-2 protein stimulus on the cells in addition to osteogenic medium. G3 represented the influence of just the tested membrane on the cells. G4 represented the AMOR strategy in the membranes, and G5 demonstrated whether the absence of antibody immobilization impacted expression of the evaluated genes.

**Fig 7:**
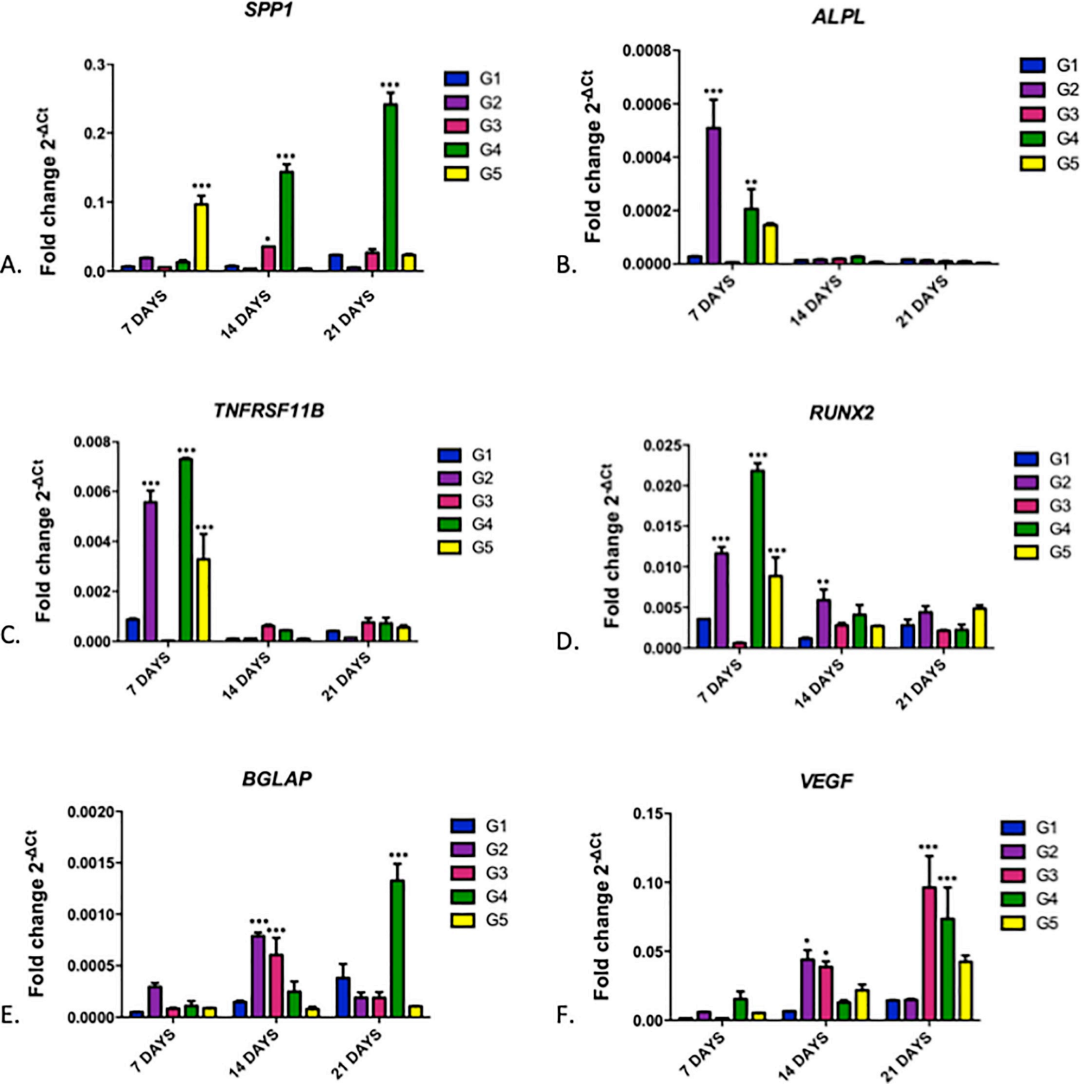
Quantitative RT-qPCR analysis of cultured MC3T3-E1 cells after G1, G2, G3, G4 and G5 group treatments over 7, 14, and 21 days, demonstrating gene expression of *SPP1* (A), *ALPL* (B), *TNFRSF11B* (C), *RUNX2* (D), *BGLAP* (E), and *VEGF* (F) genes normalized by the *GAPDH* gene. Bars indicate mean gene expression (2^-ΔCt^) +/- standard error. Two-way ANOVA followed by the Bonferroni Test. *** = p <0.001; ** = p <0.1, * = p <0.05, compared to the control group (G1) for each period.

Gene expression analysis was assessed from MC3T3-E1 cells cultured in osteogenic medium with the treatments shown in Table 1 over 7, 14, and 21 days. Fig 7 shows quantitative RT-qPCR analysis of gene expression for *SPP1* (7A), *ALPL* (7B), *TNFRSF11B* (7C), *RUNX2* (7D), *BGLAP* (7E), and *VEGF* (7F) genes normalized by the *GAPDH* gene.

Candidate gene expression showed an overall significant increase in G4 (MC3T3-E1 + BC-HA-anti-BMP-2 + 200 ng/mL Protein) during the analyzed periods which may suggest an acceleration in osteoblast differentiation/activity using this biomaterial.

*SPP1*, *BGLAP* and *VEGF* genes showed increased gene expression proportional to contact period with cells in G4; this was more evident and significant at 21 days compared to the other groups (Fig 7B, 7C and 7D).

*ALPL*, *RUNX2* and *TNFRSF11B* genes demonstrated increased gene expression in G4 within 7 days; this difference was not sustained in the following periods (Fig 7A, 7E and 7F).

G4 was of high interest as it simulated a situation where BC-HA-antiBMP-2 was in contact, in an in-vivo environment, with endogenous BMP-2 in a bone repair process; *ALPL*, *RUNX2* and *TNFRSF11B* genes showed early increased gene expression, whereas *SPP1*, *BGLAP* and *VEGF* genes showed increasing expression with increasing biomaterial contact time.

### Detection of mineral nodules formation by alizarin red

Table 1 shows the groups of mineral nodule analysis on each material after 7, 10, and 14 days of MC3T3-E cell culture.

**Fig 8:**
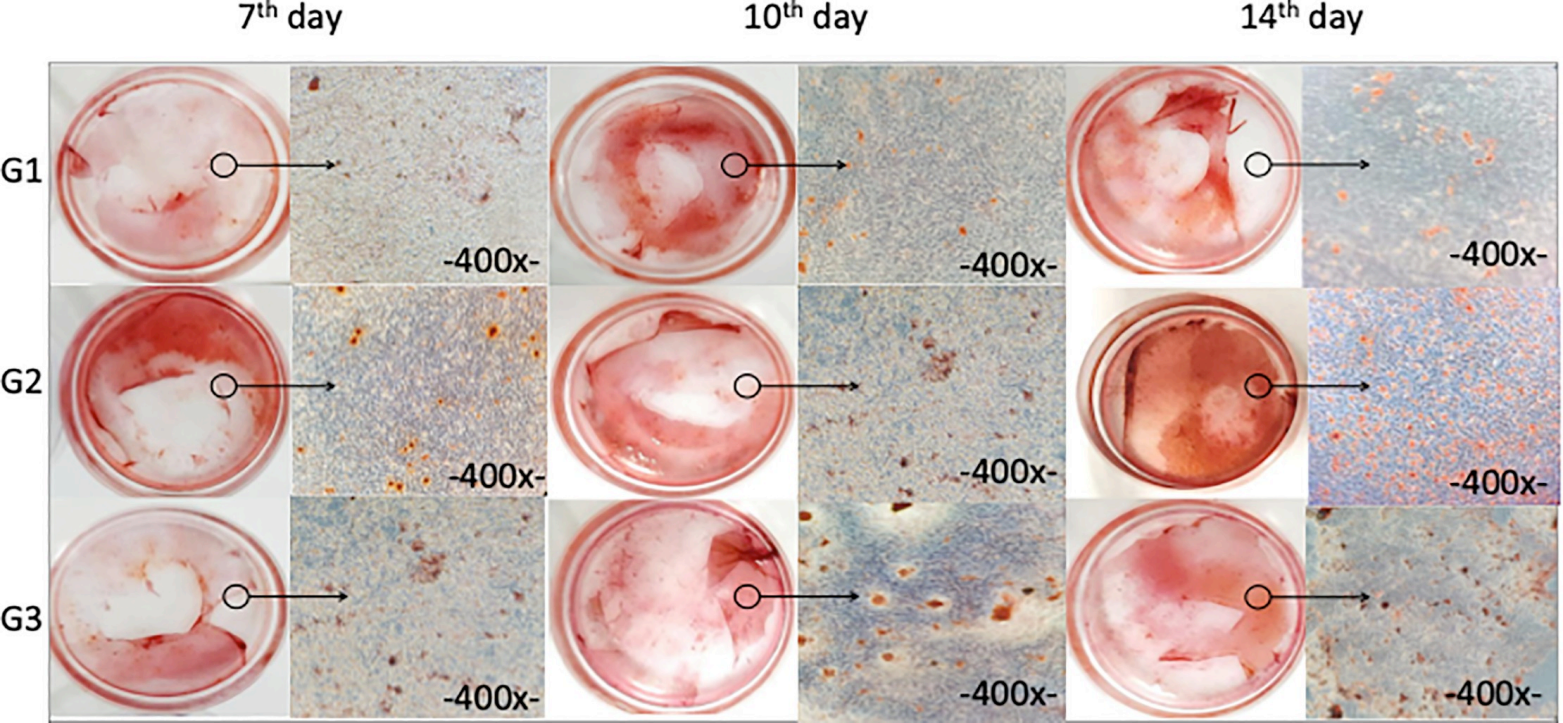
Macroscopic and microscopic analysis of mineral nodules by Alizarin Red in groups G1, G2, and G4 after 7, 10, and 14 days of cell culture.

**Fig 9:**
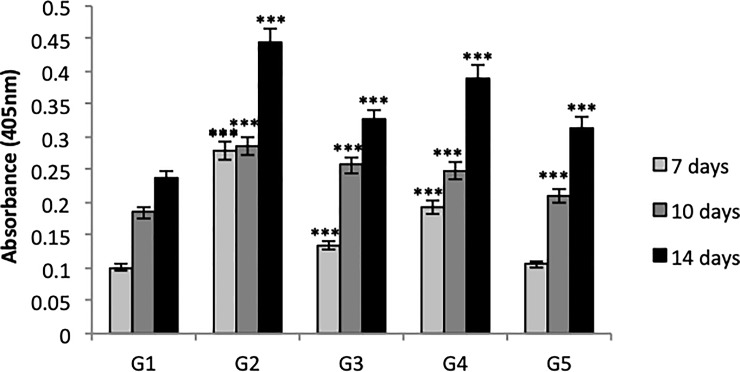
Quantitative analysis of mineral nodules by Alizarin Red in G1, G2, G3, G4, and G5 at 7, 10, and 14 days. Bars indicate the highest osteogenic potential of cultures according to investigated material +/- standard error. One-way ANOVA followed by Tukey's test. *** = p <0.001 compared to the G1 control group.

The control materials (G1 and G2) and material of interest (G4) were macroscopically and microscopically evaluated (Fig 8). Quantitative analysis of mineralization was evaluated in all groups (Fig 9).

Mineral nodule formation significantly increased in all groups compared to G1. This indicates that the presence of some external factor such as BC-HA, BMP-2 protein, and isolated or combined anti-BMP-2 antibody are able to accentuate osteogenic potential.

The groups treated with BMP-2 protein (G2, G4 and G5) demonstrated a greater rate of nodule formation at the end of 14 days. Knowing that G2 was positive control, G4 (MC3T3-E1 + BC-HA-anti-BMP-2 + 200 ng/mL cells), the group of interest and an alternative treatment for G2, produced a satisfactory result and shows promise as a biomaterial for use in bone repair.

### ALP activity

**Fig 10:**
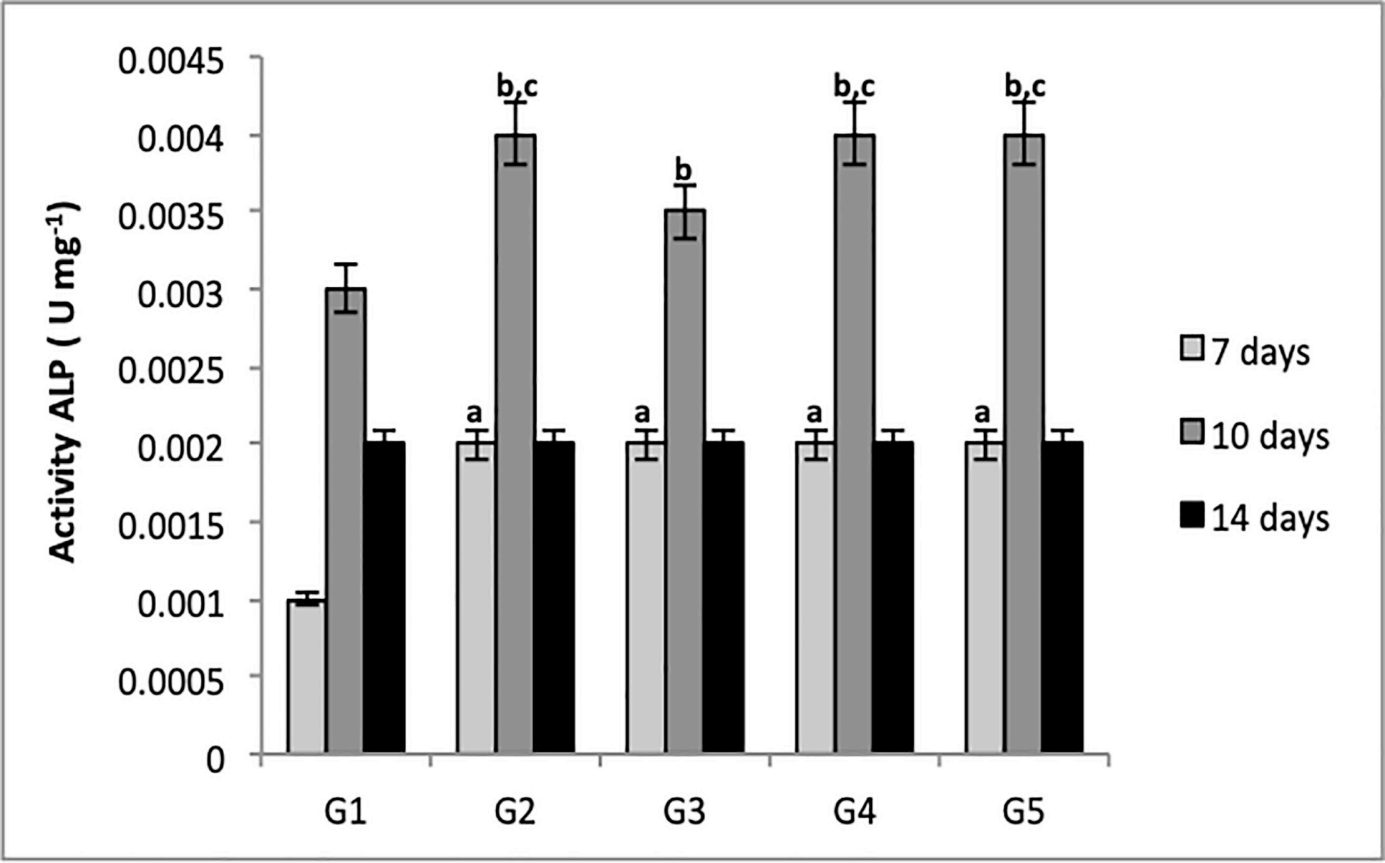
Quantitative analysis of ALP activity in G1, G2, G3, G4 and G5 at 7, 10, and 14 days at 595 nm. Bars indicate ALP activity according to group +/- standard error. One-way ANOVA followed by Tukey's test. Letters indicate statistically significant differences; a = p<0.001 compared to G1 (control group—7 days), b = p<0.001 compared to G1 (control group—10 days), c = p<0.001compared to G3.

ALP protein activity was also verified on the materials (Table 1) after MC3T3-E1 culture at 7, 10, and 14 days. Results are expressed as the ratio of ALP concentration to total protein quantity (Fig 10).

There was a significant difference in all groups compared to G1 at the 7^th^ and 10^th^ days. It can be concluded that ALP activity can also be influenced by an external factor.

All groups displayed an increase in ALP activity at day 10, indicating that its activity occurs between 8–12 days, followed by a decline.

These data agree with the result presented in ALP gene expression, indicating its increased expression near the 7^th^ day followed by a decline. Gene expression assay also confirmed that G2, G4, and G5 exhibited relatively increased ALP gene expression by about 10 days; this may be justified by the use of BMP-2 protein in these groups.

## Discussion

We investigated the physical-chemical characteristics of the developed BC-HA membranes. FTIR analysis of the functional groups related to precipitated apatite phases in the membranes revealed that HA was efficiently incorporated into the BC-HA membrane (Fig 2). The main attributes that characterize cellulose were maintained and a possible interaction between HA crystals and cellulose hydroxyl groups was suggested by the decrease in intensity of the 3500–3200 cm^-1^ band. In the BC-HA spectrum, the decrease in intensity seen in this band can be directly related to the hydrogen bonds between the hydroxyl groups and apatite phases. The chemical bond between HA and BC stabilized the composite and could maintain the mechanical integrity necessary for bone replacement [15,16].

Morphology and structure of the composites, as well as analysis of the incorporated apatite crystals were evaluated by MEV/EDS (Fig 1). Micrographs for BC-HA membrane revealed the presence of nanometric HA crystals covering the BC fibers. Altough BC has relatively small pores of nanometric order, they allow ions to freely enter the matrix, favoring the formation of HA crystals into its three-dimensional structure [16]. The molecular structure of BC may have contributed to the homogeneous deposition of apatite crystals, as previous studies have found that functional groups, such as hydroxyls, phosphates and carboxylic acids, favor apatite phase nucleation [15,25,26]. The cellulose hydroxyl groups have strong negative dipoles which promote the binding of free Ca^2+^ cations of CaCl_2_ solution and form coordinated bonds. Thus, PO_4_^3-^ ions bind to the calcium associated with BC forming the initial nucleus to form the precursor phases of apatite [21, 26, 27].

EDS analysis revealed that Ca/P molar ratios were measured and for BC-HA membranes a Ca/P molar ratio of 1.52 was obtained for calcium deficient hydroxyapatite (CDHA) [28] (Fig 1E). This decrease in Ca/P molar ratio may be due to the initiation of Ca^2+^ and PO_4_^3-^ ion dissolution, the replacement of Ca^2+^ ions by Na^+^, or the substitution of PO_4_^3-^ ions by CO_3_^2-^ [16].

The mechanical properties of the membranes were evaluated by tensile strength, elongation and modulus of elasticity (Table 2). All samples containing HA were shown to be less mechanically resistant (lower Rp values) and significantly different to membranes without HA. The presence of HA may have been responsible for the formation of a highly compacted structure with small intermolecular spaces. This may not have allowed a sufficient structural rearrangement due to plastic deformation, reaching the yield point faster and characterizing a more fragile structure [29].

The membranes without incorporated HA, on the other hand, were more mechanically resistant, probably due to the formation of a less compacted structure with large intermolecular spaces. This may have allowed an extensive structural rearrangement under the application of tension before yield point was reached. BC membrane Ep and Rp results are in agreement as membranes exhibiting the highest energy expenditure in perforation also showed higher Rp. Evaluated parameters increased in moistened samples revealing that membranes became less fragile. These results may be interesting as they suggest greater in vivo membrane resistance. However, Saska et al.,[16] reported that bioceramics have a limitation in clinical use, due to their low mechanical resistance in comparison to other metallic and polymer biomaterials. This does not allow them to be used as anchorage for implants, but as coatings for high mechanical resistance materials, or as bone filling materials [16].

Sepúlveda et al., [30] showed that HA cement facilitates bone contour and is clinically used as a bone restorative bioceramic because of its similarity to the bone mineral composition and its osteoconduction capacity, being an important biomaterial for bone replacement in orthopedics, craniofacial reconstructive surgery, dentoalveolar implant, and oral surgeries [16].

The hydrophilic properties of the composites were evaluated by the contact angle test both before and after immobilization of anti-BMP-2 antibodies on the membranes (Table 3). All materials were shown to be hydrophilic because they had a contact angle θ<90 °. The presence of antibodies in the samples reduced this angle even further. The membranes became more hydrophilic, which is beneficial for cell adhesion and proliferation. These results may have been due to the presence of polar groups of antibodies which provide a higher surface energy thus reducing water surface contact angle [31].

Vellayappan et al., [31] characterized nanosilver-impregnated silsesquioxane/poly (carbonate-urea) polyester urethane polymer (POSS-PCU-NS) with covalently bound anti-CD34 antibodies to improve the capture of endothelial progenitor cells (EPC) with antithrombogenic potential. The POSS-PCU polymer exhibited a certain degree of hydrophobicity due to its higher contact angle. Water contact angle results showed that the surface of POSS-PCU became more hydrophilic after antibody binding on the sample surface. Chong et al., [28] investigated the capture of endothelial progenitor cells by poly (CD34) conjugated on the surface of polycaprolactone (PCL) films for cardiovascular implant applications in order to promote endothelialization. Contact angle measurements demonstrated that the surface of the PCL film is highly hydrophobic (73.5°±3.7°) and the subsequent conjunction of CD34 antibodies caused a statistically significant decrease in hydrophobicity (65.2°±2.7°), again agreeing with our results.

The use of natural polymers for biomaterials has advanced in recent years due to the interesting physicochemical properties they offer. However, according to Singh et al.,^.^[18] indirect DNA damage such as promoting oxidative stress and inflammatory response may also occur due to the possible interaction between free particles in the cytoplasm and DNA during the cell division process. Tests are therefore needed to verify their non-toxic potential and safety for use as biomaterials.

BC and BC-HA membranes were evaluated in vitro to ensure the non-toxic potential in the MC3T3-E1 cell line, a pre-osteoblastic lineage. Cytotoxicity assessed by XTT assay was evaluated over 7, 14, and 21 days (Fig 3). Each test period showed cellular viability above 80%, indicating the non-cytotoxic character of the membranes.

The 7^th^ day result showed a slight decrease in cell viability compared to the other periods (14 and 21 days). Kim et al., [32] associated this fact with the extracellular adhesion proteins, responsible for adering the cells to the biomaterial and allowing their proliferation. Regarding the assay in MC3T3-E1, there could have been an initial disturbance in the system during the cell to membrane adhesion phase which was subsequently solved, as can be seen at 14 and 21 days. Thus, the non-cytotoxic character of BC-HA was confirmed.

He et al., [33] and Tazi et al., [34] verified the non-toxic character and biocompatibility of BC associated with HA in an organic-inorganic film in 293T cells; and scaffolds in Saos-2 osteoblastic lines, respectively, using MTT assay. According to their results and those in our study, it is possible to conclude that BC-HA materials, independent of assay and cell lineage, were not cytotoxic and have been shown as excellent matrix for anti-BMP-2 incorporation.

Massari et al., [35] studied the tissue response in vivo after subcutaneous implantation of BC and hydroxyapatite (Hap) membranes in male rats. The number of mononuclear and polymorphonuclear cells, and the presence of fibrous capsule were evaluated. They found no difference in the inflammatory pattern of BC and BC-Hap materials compared to the polytetrafluoroethylene standard, which agree with our vitro results.

Scarel-Caminaga et al., [36] developed BC membranes functionalized with HA or collagen with or without osteogenic growth peptides (OGP) as a promising biomaterial for bone repair. They evaluated material toxicity using the same tests as ours in CHO-K1 cells. Our results are in agreement with theirs, which demonstrated the absence of cytotoxicity, genotoxicity and mutagenicity in BC-HA membranes.

Antibody adsorption on the membranes was confirmed by in vitro study using a mouse anti-human secondary antibody conjugated to FITC (Fig 6). There was a reduction in antibody adsorption of approximately 70% in 7 days and 90% in 14 days. Ansari et al., [37] verified the binding and release kinetics of anti-BMP-2 monoclonal antibodies associated with a complex with G protein on an absorbable collagen sponge (ACS) for up to 14 days. Results obtained by secondary antibodies associated with FITC confirmed antibody binding in the ACS. The presence of G protein used to increase the binding of anti-BMP-2 monoclonal antibodies in the scaffolds did not inhibit antibody release. In addition, no statistically significant differences were found in the levels of antibodies detected in the ACS + protein G scaffold or ACS only scaffolds during the evaluated period (1,7 and 14 days).

Moshaverinia et al., [38] studied the binding and in vitro release of anti-BMP-2 monoclonal antibodies (25 μg/mL) in alginate microspheres. Similar to our study, the antibody release profile of the alginate microspheres was evaluated using fluorescence microscopy with fluorophore-coupled secondary antibody (FITC) and also showed antibody uptake for up to 14 days.

Gene expression analysis revealed increased expression of *SPP1*, *BGLAP*, *VEGF*, *ALPL*, *RUNX2* and *TNFRSF11B* candidate genes in G4 (MC3T3-E1 + BC-HA-anti-BMP-2 cells + 200 ng/mL BMP-2 protein), some being earlier and others later than the analyzed period. In Fig 7B, expression level in G2 was higher than G4, demonstrating that BMP-2 protein alone is capable of promoting gene expression in the repair process, which is indeed correct. The main role of BMPs is the recruitment of mesenchymal cells to the healing site and then differentiating them into osteogenic lineage, resulting in new bone formation. In addition, BMP-2 plays an important role in the expression of osteogenic markers such as alkaline phosphatase (ALP) [2]. However, it is known that to achieve the expected result, supra-physiological doses are required, which have a high cost, plus the inability to maintain the growth factor concentration long periods and the lower biological activity of rhBMPs compared to endogenous BMP [14].

Pigossi et al., [39] investigated the bone repair potential of a BC-HA composite with and without osteogene growth peptide (OGP) in mice through the expression of *ALPL*, *RUNX2*, *TNFRSF11B*, *SPP1*, *BGLAP* and *VEGF* genes. Similar to our study, *RUNX2* and *TNFRSF11B* genes showed significant increases during early bone regeneration in the presence of BC-HA-OGP. The other genes were expressed in the later bone regeneration periods, with statistical differences compared to the control group.

He et al., [40] developed a scaffold based on chitosan, alginate, and hydroxyapatite (QAH) as a BMP-2 transporter and evaluated in vitro gene expression in mesenchymal cells. The osteponin and osteocalcin genes were evaluated for QAH scaffold with and without BMP-2, and results demonstrated that the presence of BMP-2 significantly increased the expression of these genes, in the same way as results in our study. The authors concluded, in conjunction with other in vitro and in vivo assays, that the combination of QAH/BMP-2 improves bone formation and promotes an almost complete repair of calvarial defects in mice.

Guex et al., [41] investigated the gene expression of *ALPL*, *RUNX2* and collagen genes in a scaffold based on poly (3,4-ethylenedioxythiophene) polystyrene sulfonate (PEDOT: PSS) for 28 days in MC3T3-E1. Similar to our results, they found that all genes demonstrated increased expression compared to the amount expressed on the first day, revealing their importance in the bone repair process by osteogenic precursor cell (MC3T3-E1) differentiation and mineralization deposit.

Calasans-Maia et al., [42] studied nanostructured carbonated hydroxyapatite/calcium alginate (HCA) microspheres used for alveolar bone repair. They investigated RANK/OPG levels as it has been described as a dominant mediator of osteoclastogenesis; they found increased bone resorption and an initial release of molecules involved in bone activation. Our study also demonstrated an increase in *TNFRSF11B* in the initial periods, a promising indication of action from the biomaterial in bone repair.

Zaborowska et al., [43] demonstrated that nanoporous bacterial cellulose scaffolds (BC-n) facilitate the growth of osteoblasts and the formation of a mineralized tissue after 7 and 14 days in MC3T3-E1 by alizarin red mineral nodule assay. These results are in accordance with ours, where only the presence of BC-HA membrane was able to induce the formation of mineral nodules compared to controls (G1).

Luppen et al., [44] studied the role of BMP-2 (10 and 100 ng/mL) in restoring bone mineralization after treatment with glucocorticoids (GC), which inhibit the action of osteoblasts and induce osteoporosis. ALP and alizarin red assays were performed on MC3T3-E1 cells. Similar to our results, ALP activity was detected reaching a maximum level in 10 days, demonstrating its role, at both concentrations, in bone repair. The formation of mineralization nodules prevented by GC was also restored when treated with BMP-2 at both concentrations.

Our study shows that the presence of BMP-2 protein induced greater formation of mineral nodules in groups G2 and G4 and increased ALP activity in groups G2, G4, and G5 (Figs 8 and 9). The essential role of this protein has once again been demonstrated in the recruitment of mesenchymal cells to the site of healing and subsequent differentiation into osteogenic lineage, resulting in new bone formation.

The study by Zimmermann et al., [45] also in agreement with our study, demonstrated that the presence of BC and HA nanocomposites significantly increased ALP activity after 12 days in MC3T3-E1 cells, showing it to be an excellent material for bone repair (Fig 10).

## Conclusion

We can conclude that BC-HA-anti-BMP-2 membrane presented expected physicochemical characteristics. It was confirmed by the MEV-EDS and FTIR assays the presence of the BC and HA components in the membranes, obtained resistance mechanics required to act as bone filler materials and is classified as hydrophilic which allows cell adhesion and proliferation. BC-HA-anti-BMP-2 is a non cytotoxic, genotoxic and mutagenic biomaterial in MC3T3-E1 cells. The gene expression results showed that the biomaterial increases the expression of genes related to bone repair. The bone mineralization and alkaline phosphatase activity evaluation demonstrated that the biomaterial increased the mineralization nodules and the levels of ALP activity when compared to the control group.

Therefore, the bone repair process mediated by monoclonal antibodies is an alternative that must be evaluated more precisely. In vitro studies of biomaterials are important to make a global and also specific analysis, evaluating different properties, before performing in vivo studies. We know that the toxicity of a biomaterial observed in vitro tests is a signal that some modification must be done. After non toxic results in vitro tests, then in vivo evaluation will be necessary. The purpose of BC-HA-anti-BMP-2 is to act in bone repair, and this action depends on the recrutation of endogenous BMP. The first step (in vitro tests) showed good results and demonstrated tha it can be a promising biomaterial. Therefore, in vivo tests are necessary for the verification of the action of this biomaterial, and if the results will be better compared the absence of it, and if it will be a better and more economical alternative when compared to the use of BMPs.
